# Heterotrimeric G Protein-coupled Receptor Signaling in Yeast Mating Pheromone Response*

**DOI:** 10.1074/jbc.R116.714980

**Published:** 2016-02-23

**Authors:** Christopher G. Alvaro, Jeremy Thorner

**Affiliations:** From the Division of Biochemistry, Biophysics and Structural Biology, Department of Molecular and Cell Biology, University of California, Berkeley, California 94720-3202

**Keywords:** adaptor protein, cell differentiation, G protein, G protein-coupled receptor (GPCR), gene regulation, mitogen-activated protein kinase (MAPK), morphogenesis, post-translational modification (PTM), Saccharomyces cerevisiae, scaffold protein, signal transduction, gene regulation, protein phosphorylation, cell adhesion, cell cycle arrest, polarized morphogenesis, cell fusion, nuclear fusion

## Abstract

The DNAs encoding the receptors that respond to the peptide mating pheromones of the budding yeast *Saccharomyces cerevisiae* were isolated in 1985, and were the very first genes for agonist-binding heterotrimeric G protein-coupled receptors (GPCRs) to be cloned in any organism. Now, over 30 years later, this yeast and its receptors continue to provide a pathfinding experimental paradigm for investigating GPCR-initiated signaling and its regulation, as described in this retrospective overview.

## Genetic Analysis Provides an Enumeration of Molecular Parts

**FIGURE 1.**
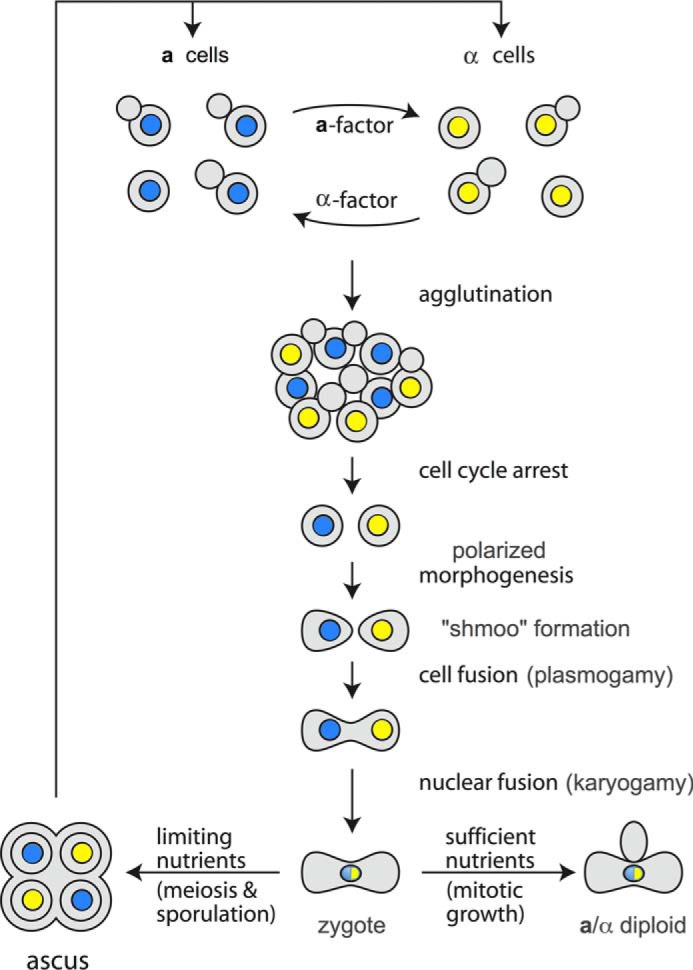
**Overview of the events that occur in pheromone-induced conjugation (“mating”) of the two haploid mating types of the budding yeast *S. cerevisiae*.** Adapted from Ref. 100. © 2006 Cold Spring Harbor Laboratory Press.

Despite its deceptively simple lifestyle as a unicellular microbe, *Saccharomyces cerevisiae* (bakers' yeast) exists in three distinct cell types. There are two haploids, termed **a** cells and α cells. The third cell type, an **a**/α diploid, is formed by the pheromone-induced conjugation or “mating” of an **a** and an α cell, just as the fusion of any two, compatible haploid gametes (*e.g.* sperm and egg) forms a diploid zygote (Fig. 1). The distinction between an **a** cell and an α cell is controlled by two non-homologous alleles (dubbed *MAT***a** and *MAT*α) at a single locus located slightly centromere-proximal from the middle of the longer arm of Chromosome III (1). Analysis of the DNA sequence, transcripts, and polypeptides encoded at the *MAT***a** and *MAT*α loci demonstrated that these sequences encode transcriptional activators and repressors that, along with additional factors for co-activation and co-repression, control expression of the genes that confer the characteristics of the two different haploid cell types (and of the diploids arising from their mating) (1).

The genes responsible for mating behavior that are controlled by the regulatory proteins encoded at *MAT* were largely identified in the search for and characterization of non-mating (“sterile” or *ste*) mutants, *i.e.* a rare **a** cell that grows normally, but is unable to mate with an α cell (and vice versa) (2). Conceptually speaking, there should be two basic classes of *ste* mutants: (i) those that are defective in producing their cognate pheromone (so that they cannot stimulate a partner), but are still able to respond to the pheromone of the other mating type; and those that are able to produce their cognate pheromone, but are defective in responding to the pheromone of the other mating type. The latter are more informative for delineating the gene products required by a haploid cell for transducing its exposure to a pheromone into appropriate downstream physiological responses. Subsequent genetic interrogation of the *ste* mutations (segregation analysis, mapping, complementation tests and, with the advent of recombinant DNA technology, eventually cloning of the corresponding DNA), followed by elucidation of the biochemical properties of the *STE* gene products, generated a rather comprehensive catalog of the functions involved in the production and response to pheromone. Gene products with overlapping functions (*e.g.* two MAPKs, Fus3 and Kss1), as well as factors necessary for mating, but essential for growth (*e.g.* the small GTPase Cdc42), are required for pheromone response, but were identified by other means. Similarly, mutants defective in executing distal steps in mating, such as cell fusion (3) and nuclear fusion (4), have been isolated, and the cognate genes have been characterized. From this genetic parsing of parts and from examination by numerous researchers in this field of the corresponding proteins and their interactions, order of function, subcellular localization, state of post-translational modification, abundance, stability, and the effects of various perturbations (both modeled mathematically and assessed experimentally using innovative new tools, including single-cell analysis and microfluidic devices), has emerged a remarkably in-depth and informative picture of the events required for the mating process.

## Yeast Pheromone Receptors

*STE2* and *STE3* were the first genes for agonist-binding GPCRs^3^ to be cloned and characterized (5). Ste2 is the GPCR in the plasma membrane (PM) of *MAT***a** cells that recognizes the pheromone made by *MAT*α cells (α-factor, a 13-residue peptide with unblocked N and C termini). The features of α-factor required for binding to its orthosteric site and for activation of Ste2 have been explored by examining structure-activity relationships in peptide agonists and antagonists derived by genetic methods (6) and by chemical synthesis (7). Similarly, the properties of the receptor and the residues involved in its folding, membrane trafficking, and ligand discrimination have been extensively studied by both targeted and unbiased mutagenesis (8–10). As for other GPCRs, there is some evidence that Ste2 function can be influenced by compounds binding to allosteric sites (11, 12). Genetic data (13), FRET analysis (14), Cys mutagenesis and disulfide cross-linking (15), and other evidence (16) indicate that Ste2 functions as an oligomer. Ste3 is the GPCR in the PM of *MAT*α cells that recognizes the pheromone made by *MAT***a** cells (**a**-factor, a 12-residue lipopeptide with an unblocked N terminus and an *S*-farnesylated and methyl-esterified C-terminal Cys). Because of the relative insolubility of its cognate ligand, analysis of Ste3 has lagged behind studies of Ste2.

Remarkable advances have been made during the last decade in stabilizing, purifying, crystallizing, and determining the structures of GPCRs by x-ray diffraction, including, as of this writing, structures for 10 peptide-binding receptors in the so-called Class A family of GPCRs and two peptide-binding receptors in the so-called Class B. However, despite valiant attempts to purify a C-terminally tagged version of native Ste2 (11), otherwise engineered variants (17), and a Cys-less and non-glycosylated derivative (18), the structure of this yeast GPCR remains unsolved, although the conformational behavior of large fragments of Ste2 has been examined by NMR (19).

## GPCR-initiated Signal Propagation

**FIGURE 2.**
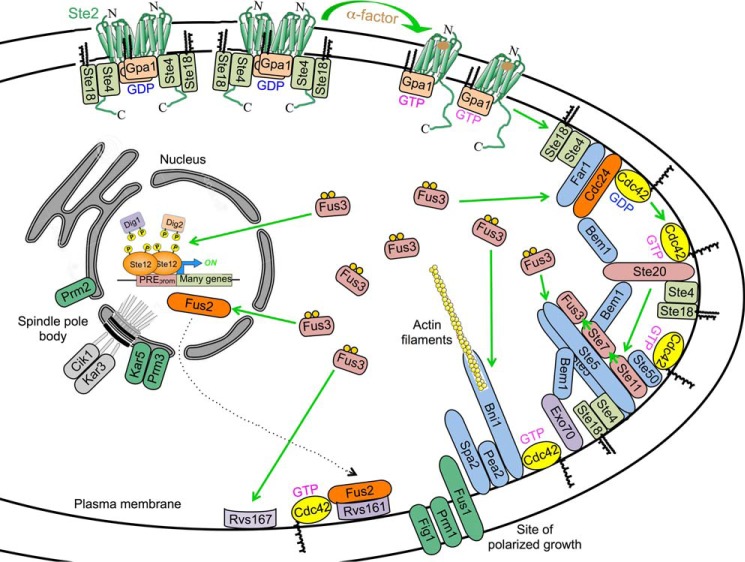
**Schematic representation of the GPCR-initiated biochemical processes required for execution of the mating pheromone response program.** For clarity, activation and roles of the ancillary MAPK Kss1 have been omitted. Whether pheromone binding causes dissociation of Ste2 dimers concomitant with GTP-for-GDP exchange in Gpa1 (Gα) and release of free Ste4-Ste18 (Gβγ) is a speculative model. See text for further details.

Genetic studies and biochemical analysis have demonstrated that Ste2 and Ste3 are coupled to the same heterotrimeric G protein, composed of Gpa1/Scg1 (Gα) and Ste4-Ste18 (Gβγ), and engage the same downstream components. The accompanying schematic diagram (Fig. 2), showing the response of a *MAT***a** cell to α-factor, is an attempt to capture the conformational changes and dynamics, both temporally and spatially, of these events, but no single static and two-dimensional illustration can do so adequately. Upon ligand binding, Ste2 undergoes a conformational change that allows it to act as a guanine-nucleotide exchange factor (GEF) on Gpa1. Replacement of GDP with GTP in Gpa1 causes a conformational change that dissociates the Gβγ complex (Ste4-Ste18). This system was the first in which it was unequivocally demonstrated that a released Gβγ complex serves as the critical positive effector for initiating downstream signaling. In cells expressing a Ste4-Gpa1 fusion protein, downstream signaling is activated in response to pheromone just as effectively as when Ste4 and Gpa1 are expressed as separate proteins (20). Hence, as long as GTP-bound Gα no longer occludes its interface with Gβγ, full dissociation of the heterotrimer is not required for pathway activation. In this regard, *N*-myristoylation (C14) of Gly^2^ and *S*-palmitoylation (C16) of Cys^3^ in Gpa1 and *S*-palmitoylation of Cys^206^ and *S*-farnesylation (C15) and methyl esterification of the C-terminal Cys^207^ in mature Ste18 (Gγ), tightly associated with Ste4, will firmly anchor such a Ste4-Gpa1 chimera at the PM. Hence, positive signaling roles ascribed to endocytosis of Gpa1 and its interaction on the surface of the vacuole with the PtdIns 3-kinase Vps34 (21) can only have, at best, a minor role in the early processes that initiate mating. By contrast, positive signaling roles ascribed to the interaction of Gpa1 with effectors that productively execute their functions in mating at the PM, such as direct association of Gpa1 with the MAPK Fus3 (22), which phosphorylates a number of PM-associated targets (see below), and interaction of Gpa1 with the polysome-associated and KH domain (an RNA recognition motif)-containing protein Scp160 (23), which binds to mRNAs encoding PM-localized cell polarity proteins needed for the anisotropic growth leading to shmoo formation (24), seem of significantly greater biological importance. Nonetheless, as initially revealed genetically, the primary role of Gpa1 is to negatively regulate signaling by occluding the effector-binding surface of Ste4 in the Gβγ complex.

Freed Gβγ evokes three synergistic processes that must act in concert with each other to activate the downstream MAPK cascade successfully. All occur at the PM because Gβγ is firmly tethered there by virtue of the aforementioned lipophilic modifications of Ste18 to the penultimate residue (Cys^206^) and Cys^207^ of its C-terminal C*AAX* box (where C represents a Cys residue, *A* represents any aliphatic residue, and *X* represents any residue), -NSNSV**C**^C16^**C**^C15^-C=O-OCH_3_ (25). First, free Gβγ recruits the scaffold protein Far1, along with its interaction partner Cdc24, which is the GEF for the small (21.3-kDa) GTPase Cdc42 (26). The Far1·Cdc24 complex shuttles in and out of the nucleus, but is found predominantly inside the nucleus in naive cells; however, after cells are exposed to pheromone, the presence of free Gβγ allows for the capture and accumulation of Far1·Cdc24 at the PM (27). Both Far1 and Cdc24 contain phosphoinositide-binding PH (pleckstrin homology) domains, which further stabilize their PM binding. Cdc24 also associates with a multi-purpose linker protein, Bem1, via interaction of their respective C-terminal PB1 domains (28). The substrate of Cdc24, Cdc42, is also firmly tethered at the PM by *S*-geranylgeranylation (C20) and methyl esterification of the C-terminal Cys in its C*AAX* box, -IKKSKK**C**^C20^-C=O-OCH_3_, and by the adjacent basic (Lys) residues that promote association with the headgroups of acidic PM glycerophospholipids (29). Thus, pheromone-induced propinquity of Cdc42 with its GEF generates a localized pool of active (GTP-bound) Cdc42.

Second, a direct target of GTP-Cdc42, the Cdc42-activated protein kinase Ste20, the first eukaryotic p21-activated protein kinase (PAK) identified, is also accumulated in the same vicinity of the PM because it contains a specific Gβγ-binding (GBB) domain at its C-terminal end, which is conserved in mammalian PAKs (30), as well as a novel basic residue-rich PtdIns(4,5)P_2_-binding element (31). Purportedly, Bem1 can also bind to Cdc42 (32), but this interaction is not required for PM recruitment of Cdc24 (33). However, two N-terminal SH3 (Src homology 3) domains in Bem1 bind to Pro-*X-X*-Pro sites in Ste20 (34), thus helping to bring the machinery that catalyzes Cdc42 activation into juxtaposition with Ste20, thereby generating a localized pool of the active state of this protein kinase. A primary substrate of Ste20 is the MAPKKK Ste11 (35). Thus, Ste20 serves as a MAPKKKK.

Third, in response to pheromone, Ste11 is also restricted to the PM for two reasons. Ste11 associates tightly with a small non-catalytic subunit, Ste50, via interaction of their respective N-terminal sterile alpha motif (SAM) domains (36), and the C-terminal so-called RA (Ras association) domain of Ste50 associates preferentially with Cdc42-GTP (not Ras-GTP) (37). However, the majority of the Ste11·Ste50 heterodimer is bound to the scaffold protein Ste5, which also has binding sites for the MAPKK Ste7 and the MAPK Fus3 (38), and likely functions as an oligomer (39). Like Far1·Cdc24, the complex of Ste5 and the MAPK cascade components undergoes continuous nucleocytoplasmic shuttling and is found mainly inside the nucleus in naive cells; however, after pheromone administration, interaction of free Gβγ with the RING-H2 domain of Ste5 (40) allows for capture and accumulation of the Ste5 complexes at the PM (41). In addition to its interaction with Gβγ, association of Ste5 with the PM is further stabilized by an N-terminal, phosphoinositide-binding, basic amphipathic α-helix (PM motif) (42) and by an internal PH domain (43). There is also evidence that Bem1 binds to Pro-*X-X*-Pro sites in Ste5 (44), and it also contains a phosphoinositide-binding PX (Phox homology) domain (45), all of which would further contribute to drawing all the necessary components into close proximity at the same region of the PM, which has been shown to be highly enriched in PtdIns(4,5)P_2_ (46).

Thus, to fire the MAPK cascade efficiently, pheromone must bind to a sufficient number of receptors to release enough molecules of free Gβγ to allow for the coincident localization and encounter of three different types of multi-protein complexes. This convergence establishes a meta-stable “factory” to propagate multiple rounds of sequential phosphorylation through the MAPK cascade (47). In this regard, Ste7 possesses a high-affinity docking site for Fus3 (48), and association of Fus3 with Ste5 causes Fus3 to adopt a conformation that promotes its efficient Ste7-mediated phosphorylation (49). Once activated by its dual phosphorylation (Thr(P)-180 Tyr(P)-182), the MAPK Fus3 dissociates from Ste5 (50). The pool of activated Fus3 phosphorylates a variety of targets at the PM, in the cytosol, and in the nucleus, all of which are necessary to execute the functions required for mating; the spatial and temporal dynamics of its actions are important because restricting Fus3 to either the PM or the nucleus markedly impairs mating proficiency (51).

## Outputs of the GPCR-initiated MAPK Cascade

Activated Fus3 first contributes to steps necessary for efficient mating by phosphorylation of pre-existing proteins. Fus3 imposes G_1_-specific growth arrest by phosphorylating Far1 and converting it into an inhibitor of the G_1_ cyclin (Cln)-bound form of CDK1 (Cdc28) (52), thereby synchronizing haploid cells at the same stage of the cell cycle.

Fus3 also stimulates processes that promote the highly polarized morphogenesis involved in conjugation tube extension (“shmoo” formation). Fus3 phosphorylates and stimulates the formin Bni1 required for polymerizing the actin filaments that direct secretory vesicles to the growing shmoo tip (53); Bni1 is tethered at the cell cortex by activated Cdc42 (54) as well as by cell polarity determinants (including Spa2 and Pea2) (55). After tracking along actin filaments, polarized targeting and tethering of post-Golgi secretory vesicles to active sites of exocytosis prior to their SNARE-dependent fusion are mediated by the exocyst complex, and a PtdIns(4,5)P_2_-binding exocyst subunit, Exo70, associates intimately with Bem1 (56), thereby ensuring vesicle delivery and localized membrane growth at the shmoo tip. This polarized morphogenesis also seems to be aided by Fus3-mediated phosphorylation of Ste5 (57). Because *S. cerevisiae* lacks any motility mechanism, this directional chemotropic growth allows responsive haploid cells separated even by as much as 20 μm to make contact (58).

In addition, Fus3 evokes steps necessary to achieve cell fusion. Fus3 phosphorylates and blocks the endocytosis-promoting function of the N-BAR domain-containing amphiphysin Rvs167 (59), thereby also freeing its partner N-BAR amphiphysin Rvs161 to interact with Fus2, an alternative, pheromone-induced N-BAR domain-containing protein (60). Fus2 is also either a Cdc42 GEF or a Cdc42 effector that localizes to the site of cell-cell fusion and is essential for the completion of plasmogamy (61). Fus2 becomes available in the cytosol because its Fus3-mediated phosphorylation in the nucleus promotes its nuclear export (62). Equally as important, in the nucleus, Fus3 also phosphorylates the Dig1/Rst1 and Dig2/Rst2 proteins (63) alleviating their repression of the DNA-binding transactivator Ste12, which Fus3 also phosphorylates and stimulates (64), thereby activating transcription of numerous genes (65). *FUS3* itself is such a gene, providing the means for autocatalytic reinforcement of the responsive state.

New gene products synthesized as the result of Fus3-mediated transcriptional induction are required to execute subsequent mating-specific processes. For example, two glycosylphosphatidylinositol-anchored proteins, Aga1 and its paralog Fig2, are disulfide-linked to and present on the cell surface an adhesion protein (Aga2), which enhances agglutination between **a** and α cells (66). Afr1, an apparent auxiliary subunit for yeast phosphoprotein phosphatase-1 (Glc7), brings this enzyme to the site where the mating projection develops because it binds to septin Cdc12 (67), and the septin filaments found at the bud neck in mitotic cells undergo a striking reorganization to permit shmoo formation (68) (but whether Afr1·Glc7 is catalytically active and, if so, its specific substrates, are not known). PM insertion of Fus1 (a heavily *O-*glycosylated, PM-localized, single-span transmembrane protein) (69), as well as Prm1 (a polytopic PM protein) (70) and Fig1 (a PM tetraspanin) (71), is required for fusion of the haploid cells. Similarly, insertion into the outer nuclear envelope of Kar5 (72) and Prm3 (73) (interacting integral membrane proteins), as well as Prm2 (another tetraspanin) (70), is required for fusion of the two haploid nuclei. Likewise, up-regulation of Kar3 (a microtubule minus end-directed kinesin) and its specific localization to the cytoplasmic microtubules emanating from the spindle pole body by Cik1 (a pheromone-induced kinesin-associated protein) (74) are required to draw the two haploid nuclei together. Among the Ste12-dependent genes induced by pheromone is another transcription factor, Kar4, which cooperates with Ste12 to turn on genes required for karyogamy (such as *CIK1*, *KAR3*, and *PRM2*) (75).

## Negative Feedback Mechanisms That Dampen Pheromone Response at the GPCR Level

Among the products of Ste12-dependent genes induced in response to pheromone are proteins that act to squelch further signaling. These multiple negative feedback mechanisms presumably evolved to dampen prolonged mating pheromone response because its hyperactivation causes cell death (76). Thus, even in the presence of a stimulus of constant intensity, GPCR-initiated signaling can be disabled, a process referred to as adaptation, desensitization, or down-regulation. The pheromone-induced negative regulators act at many points: Msg5, a dual specificity phosphoprotein phosphatase, deactivates Fus3 (77); binding of guanine nucleotide dissociation inhibitor (GDI) Rdi1 converts Cdc42 to the off-state by preventing nucleotide exchange and extracting it off the PM by providing a hydrophobic pocket for its geranylgeranyl substituent (78); and PM-localized synaptojanin ortholog Inp52/Sjl2, a dual action phosphoinositide-specific phosphatase, catalyzes hydrolysis of PM PtdIns(4,5)P_2_ to PtdIns (79).

**FIGURE 3.**
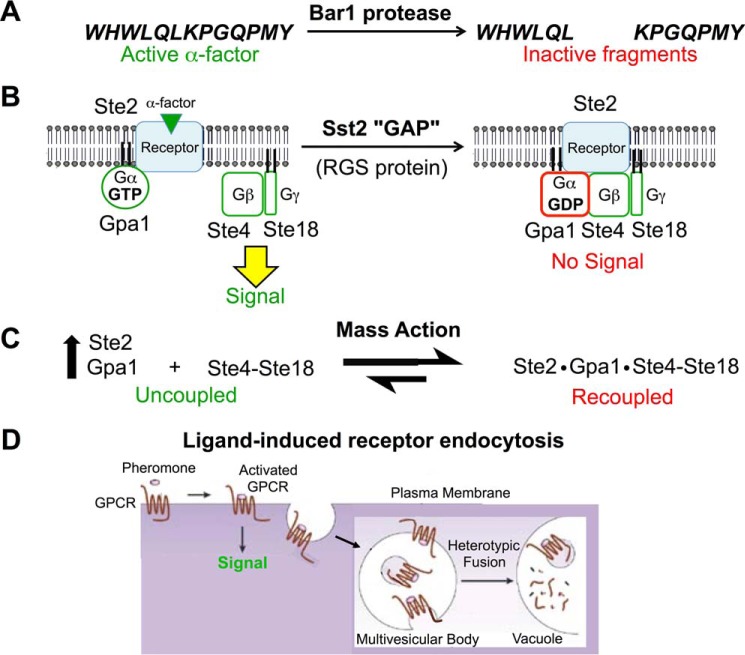
**Major negative feedback mechanisms promoting adaptation to pheromone signaling that act at the receptor level.** See text for details. *Panel D* was adapted from Ref. 97. © 2002 Nature Publishing Group.

Not unexpectedly, however, several of the induced negative regulators promote desensitization by acting on α-factor, its receptor, or the associated heterotrimeric G protein, thus preventing further GPCR-initiated signaling at its origin (Fig. 3). *BAR1* encodes a protease that cleaves α-factor into two inactive fragments (80). *SST2* encodes the first regulator of G protein signaling (RGS) identified (81). Binding of its N-terminal DEP domains to the cytosolic tail of Ste2 (82) delivers Sst2 to the PM, thereby positioning its C-terminal RGS domain to stimulate conversion of PM-localized GTP-bound Gpa1 back to its GDP state (83). Even the basal level of Sst2 expression is important for preventing spurious signaling that arises from occasional stochastic dissociation of the Ste2·Gpa1·Ste4-Ste18 complex (84). In its GDP-bound state, Gpa1 reassociates with and blocks downstream signaling by the Ste4-Ste18 complex. Recoupling and squelching of Gβγ function is further promoted by mass action (85); both *STE2* and *GPA1* (but not *STE4* or *STE18*) are up-regulated in response to pheromone (65), concomitant with an enhanced rate of *N*-myristoylation of Gpa1 (86), a post-translational modification essential for Gpa1 PM targeting, binding to Gβγ, and association with Ste2 (87).

An additional pheromone-induced negative regulator of pathway function with a less well defined mechanism of action is the fungus-specific and **a** cell-only protein Asg7. Largely genetic evidence indicates that it blocks signaling at the level of Gβγ and that it normally does so for the Gβγ produced by Ste3 (88), thus suggesting that its normal role is to ensure that, in newly formed zygotes, any signal that would emanate from Ste3 is blocked.

Finally, termination of the GPCR-mediated signal also involves ligand-induced receptor endocytosis (89). Occupancy of Ste2 by α-factor induces a conformational change in the receptor that exposes its 132-residue C-terminal tail (90), permitting, for example, Sst2 binding (82). This “open state” is made essentially irreversible by subsequent phosphorylation of multiple Ser and Thr residues in the receptor tail (91) mediated by the casein kinase I family members Yck1 and Yck2, which are anchored at the PM by virtue of dual *S*-palmitoylation of their C-terminal Cys-Cys motif via the Zn^2+^-dependent DHHC family protein:palmitoyl-CoA transferase Akr1 (92). The activated receptor is also now able to associate with two paralogous adaptor proteins of the α-arrestin family, Rod1/Art4 and Rog3/Art7 (93). These molecules interact with the receptor via their N-terminal arrestin fold domain and contain multiple PP*X*Y motifs or variants thereof (such as VP*X*Y or LP*X*Y) in their C-terminal extensions that recruit the HECT domain-containing protein:ubiquitin ligase Rsp5 (closest human ortholog is Nedd4L) via their binding to its three WW domains (94). Rsp5, which is already PM-associated via binding of its N-terminal C2 domain to acid glycerophospholipids, attaches Lys^63^-linked ubiquitin chains (95) to seven Lys residues in the Ste2 tail (82). These modifications engage the machinery for clathrin-mediated endocytosis (96) and then the endosomal sorting complexes required for transport (ESCRT) machinery that converts the endosomes to a multivesicular body, which then fuses with the lysosome-like vacuole where Ste2 and its ligand are degraded (97). In the absence of pheromone, Ste2 is removed from the PM at a basal rate via the action of a third α-arrestin, Ldb19/Art1 (93), that recognizes misfolded integral membrane proteins and thus acts as a quality control mechanism (98). Similarly, two other paralogous α-arrestins, Aly1 and Aly2, promote ligand-induced internalization of the **a**-factor receptor Ste3, and Ldb19 plays a contributory role (99). Because *S. cerevisiae* lacks any β-arrestin homolog, these findings demonstrate that α-arrestins alone are capable of promoting GPCR internalization.

## Prospectus

We anticipate that further interrogation of the network of biochemical processes required for the yeast mating pheromone response and its genetic control will continue to serve as an informative model for investigating molecular mechanisms in signal transduction and in the cell biology of stimulus-induced developmental transitions.

## Footnotes

3 The abbreviations used are:

used: GPCR: G protein-coupled receptor
GEF: guanine nucleotide exchange factor
PM: plasma membrane
PtdIns: phosphatidylinositol
PtdIns(4,5)P_2_: phosphatidylinositol 4,5-bisphosphate.
